# American prejudice during the COVID-19 pandemic

**DOI:** 10.1038/s41598-022-26163-5

**Published:** 2022-12-24

**Authors:** Christina Huber, Sasha Brietzke, Tristen K. Inagaki, Meghan L. Meyer

**Affiliations:** 1 University of California, Los Angeles, CA USA; 2 Department of Psychological and Brain Sciences, Dartmouth College, Moore Hall, HB 6027, Hanover, NH 03755 USA; 3 Department of Psychology, San Diego State University, San Diego, CA USA; 4 Department of Psychology, Columbia University, New York City, USA

**Keywords:** Psychology, Human behaviour

## Abstract

In the United States, anti-Asian sentiment has pervaded the Coronavirus 2019 (COVID-19) pandemic. Could Americans’ fear of contracting the virus relate to prejudice against Asian individuals? According to intergroup threat theory, prejudice increases toward groups of people when they are perceived as a likely cause of symbolic and/or real threat, including disease threat. We tested this perspective in the context of the COVID-19 pandemic by investigating the relationship between Americans’ concern about contracting COVID-19 and their feelings toward individuals from multiple countries. Between May 12–14 2020, participants residing in the United States (N = 932) completed an online survey assessing their (1) perceived threat of COVID-19 infection, (2) feelings of warmth and coldness toward people in America, China, Italy, Japan, and Greece, and (3) trait-level prejudice. Perceived threat of COVID-19 infection differentially related to feelings toward American and Chinese nationals and was unrelated to feelings toward people from other countries assessed. Specifically, greater threat of infection was associated with less warmth toward individuals from China, an effect moderated by trait-level prejudice. That is, participants high (but not medium or low) in trait prejudice showed a significant relationship between threat of COVID-19 infection and reduced warmth toward Chinese individuals. Threat of infection also related to greater warmth and less coldness toward American nationals, consistent with prior work indicating that disease threats amplify ethnocentrism. Collectively, results suggest that perceived threat of COVID-19 infection may correspond with prejudice toward the national outgroup associated with the disease’s origin (i.e., China), as well as national ingroup favoritism, among Americans prone to prejudice.

## Introduction

In the wake of the coronavirus disease 2019 (COVID-19) pandemic, hate crimes targeting Asian Americans surged in the United States. According to official data from police departments in 16 of America’s largest cities, anti-Asian hate crimes increased by 145% from 2019 to 2020, despite a 6% drop in hate crimes overall^1^. As of July 2020, 31% of Asian Americans reported having experienced racist slurs or jokes since the beginning of the pandemic, more than any other racial group^2^. Negative views toward Chinese people, in particular, rose in multiple western countries including the United States, in lockstep with the onset of the COVID-19 pandemic^3–6^.

This disturbing upswing in anti-Asian sentiment during the COVID-19 outbreak is likely due to a number of individual and societal factors, including psychological, historical, and geopolitical conditions^7,8^. From a psychological perspective, intergroup threat theory predicts that prejudice toward groups perceived as outgroups (i.e., a social group with which an individual does not identify^9^) increases when they are perceived as likely a cause of either a symbolic and/or real threat^10,11^. Threats of infection, particularly disease threats, are one class of threats that have been shown to amplify outgroup prejudice^12,13^. For example, perceived vulnerability to the Ebola virus disease during its outbreak in Western Africa increased Americans’ xenophobia toward West Africans^14^. These results came from a sample of participants living in the United States, where the chance of contracting Ebola was low, suggesting that the perception of a disease threat from a distant source may be sufficient to increase prejudice toward that community. Similarly, inducing disease salience in the lab (without actually contracting the disease and/or being with an infected person) increases xenophobia^15,16^, and individuals high in trait germ aversion show the greatest outgroup prejudice^17^.

Given that China was communicated as the source of the virus in American news media^18,19^, American prejudice toward Chinese nationals (here, defined as Chinese people living in China) may be particularly linked to COVID-19 threat. This possibility is supported by prior work suggesting that outgroup prejudice in response to disease threats tends to be preferential to national outgroups associated with the disease, as opposed to all national outgroups^16,20^. Moreover, Sinophobia (i.e., prejudice towards Chinese individuals) unfortunately has a long history in American culture, and disease threat has previously been used to justify harmful discrimination and violence against Chinese and other Asian American communities^7,21,22^. Collectively, these observations suggest that, at least among American participants, threat of COVID-19 infection may correspond with prejudice towards Chinese nationals. Determining this possibility is important, in part, because prejudice in response to real and symbolic threats is dangerous for recipients of prejudice^23,24^.

Previous research also finds evidence of ingroup preference in response to disease threats. For example, in American participants, individual differences in the fear of contracting diseases positively correlates with ethnocentrism, and inducing a symbolic threat of infection (i.e., viewing disease-relevant photographs) increases preference for American patriotism^15^. This line of research argues that two motivations are activated in response to disease threat: a motivation to avoid outgroup members perceived as a real or symbolic disease threat and a motivation to affiliate with ingroup members, who may be a source of support and help decrease disease spread^15,25–27^. Thus, the COVID-19 outbreak, which poses a salient threat of infection, may amplify prejudice toward outgroups associated with the origin of the virus while also increasing favoritism for national ingroups^15^.

The COVID-19 health crisis provides a unique opportunity to understand how disease threat might relate to outgroup prejudice during a real-world pandemic. To that end, we investigated whether the perceived threat of COVID-19 infection was associated with Americans’ prejudice toward Chinese nationals, as well as positive feelings toward other American nationals. Data was collected between May 12–14 2020, during which time COVID-19 was officially declared a pandemic by the World Health Organization, though case rates varied across regions of the world. COVID-19 rates in China and Italy were very high and frequently reported in the media during this time; however, China was reported as the source of the disease^18,19^. This allowed us to assess whether fear of contracting COVID-19 was associated with prejudice toward individuals from any region with high case rates, or specifically the national outgroup symbolically associated with the source of the disease (i.e., China, but not Italy). In addition to rating their concern about contracting COVID-19, participants reported on their perceptions of the typical individual living in the United States, China, Italy, Japan, and Greece. Including ratings of people in Japan and Greece allowed us to further assess whether any outgroup prejudice found for Chinese and/or Italian nationals was relatively preferential to countries with high infection rates, versus their region of the world.

To capture ingroup favoritism and outgroup prejudice, participants rated the typical person from each country on multiple scales used in prior intergroup psychology research, such as the intergroup emotion scale^28^, prejudice scale^29^, and feeling thermometer^30^. A principal component analysis identified that items on the scales tapped into two dimensions separately reflecting “warmth” (i.e., the tendency to feel positively toward and want to approach a person) and “coldness” (i.e., the tendency to feel negatively toward and want to avoid a person;^28,31^). We were therefore able to assess whether the perceived threat of COVID-19 infection was associated with feelings of warmth and coldness toward individuals in each country considered. Finally, we assessed the extent to which trait ethnocentrism and intergroup prejudice moderated results. Specifically, we predicted that individuals most prone to ethnocentrism and intergroup prejudice at the trait level may also show the strongest relationship between threat of COVID-19 infection and ingroup preference/outgroup prejudice.

## Methods

### Participants

Participants were recruited from Amazon Mechanical Turk (N = 582, 51.1% of the full sample, Males = 303, Mean age = 36.1, SD = 10.4) and Prolific (N = 557, 48.9% of the full sample, Males = 237, Mean age = 32.6, SD = 10.6). Ethnic breakdown of participants can be found in Supplementary Table 1. Sample size was determined a priori based on the need to have adequate statistical power to detect small, correlational effects between threat-of-infection and ingroup/outgroup responses, as well as available funding. Specifically, a power analysis indicated we needed a sample size of at least 853 participants to achieve 90% power to test our predictions about the relationships between threat of COVID-19 infection and prejudice toward the typical person from the countries assessed. The study was approved by Dartmouth’s and the University of Pittsburgh’s Institutional Review Boards (Dartmouth IRB 30494 and University of Pittsburgh IRB 20040131), respectively, and all methods were performed in accordance with institutional guidelines and regulations. MTurk participants received $6.25 for their participation and Prolific participants received $9.76 for their participation. These values were based on established pay rates from our labs (Meyer and Inagaki) for the two platforms. All participants provided informed consent. The data is available on Open Science Framework (https://osf.io/sa4gq/).

### Procedure

Participants completed an online survey through the platform Qualtrics between May 12 and 14 2020. Upon completing informed consent, participants answered questionnaires developed by prior research to assess prejudice at the state (e.g., feelings of warmth and coldness towards the groups assessed here) and trait (e.g., persistent feelings toward ingroups and outgroups more generally) level of analysis. To describe their state feelings toward the typical member of each national group, participants completed a 0–100 feeling thermometer^30^, the ten-item personality inventory^32^; which has been used previously to assess ‘human’ and ‘non-human’ traits in the prejudice literature^33^, the intergroup emotion scale^34^, and prejudice scale^29^ for the typical person in each country assessed (i.e., the United States, China, Japan, Italy, and Greece). To assess trait prejudice, participants also completed individual difference trait measures of ethnocentrism^35^ and intergroup disgust sensitivity^36^. To assess the perceived threat of COVID-19 infection, participants completed a questionnaire designed to assess perceived individual threat of COVID-19 infection (e.g., how concerned they were about personally contracting COVID-19^37^). This questionnaire was originally developed to assess perceived risk of contracting HIV and was revised to be specific to COVID-19 (see Supplementary Materials for more information and sample items). To assess general, trait-level concern about contracting disease, participants also completed the Perceived Vulnerability to Disease (PVD) scale^38^, a 15-item measure assessing chronic germ aversion and perceived infectability. Questionnaires were completed in a randomized order across participants. Participants were excluded from analyses if they failed to fully complete the survey (N = 148, 13%) or indicated that they were not familiar with COVID-19 (N = 59, 5.18%), leaving a final sample of 932 participants. We note this sample size exceeds the sample needed (N = 853) in order to detect small, correlational effects based on our power analysis.

At the end of the survey, participants answered demographic questions (i.e., age, race/ethnicity, gender) and were asked “How much of a public health problem is COVID-19 in each of the following countries?”, using a 1–7 scale anchored on “not at all” and “very much.” The purpose of these questions was akin to a “manipulation check”, to ensure that our participants were aware of the public health threat of COVID-19 across different regions of the world. Participants’ responses to these questions suggest they were informed regarding each country’s relative COVID-19 caseload at the time, with their scores reflecting China, Italy, and the United States’ relatively high case numbers (mean_US_ = 6.131, S.D. = 1.273, mean_China_ = 5.555, S.D. = 1.461, mean_Italy_ = 6.034, S.D. = 1.233, mean_Japan_ = 4.718, S.D. = 1.638, mean_Greece_ = 4.765, S.D. = 1.506). Indeed, China, Italy, and the United States’ ratings were each significantly greater than ratings for Japan (*t*’s > 11.641, *p*’s < 0.001) and Greece (*t*’s > 11.489, *p*’s < 0.001).

### Data analytic approach

Given that we used multiple questionnaires to assess state prejudice, we carried out a state prejudice data reduction step to minimize the number of statistical tests conducted on state prejudice scales, as well as minimize the redundancy between scale items. A principal component analysis (PCA) was run on standardized state prejudice survey responses, collapsing across all countries considered. Specifically, we ran a PCA with a varimax rotation solution. The PCA showed that responses to the prejudice scales fall into two dimensions, which we refer to as “warmth” and “coldness”. These terms were selected to best reflect the distinct affective content captured by each principal component, and to align with terminology that is often employed within the intergroup person perception literature^28,31^. Notably, two items originally designed to measure warmth–specifically, pride and pity from the intergroup emotions questionnaire^34^–fell within the coldness component in our PCA. Therefore, pride and pity were excluded from analyses to be consistent with the prior literature’s theoretical perspective that these items do not tap into coldness. See Supplementary Table 2 for a list of the items from each scale that fall into the warmth or coldness dimensions.

Next, we assessed the correlation between responses to the two trait-level prejudice scales (intergroup prejudice and ethnocentrism scales) to determine their redundancy. Trait-level intergroup prejudice and ethnocentrism were highly correlated (*r* = 0.819, *p* < 0.0001) and thus were averaged into a single “trait prejudice” score. Perceived threat of COVID-19 infection was negatively correlated with trait-level perceived vulnerability to disease (*r* = − 0.179, *p* < 0.001) and thus these two variables were examined separately in analyses. To test our predictions, we used linear mixed-effects models using R’s lme4 package^39^ and the Process & Hayes macro for moderation analysis^40^.

It is noteworthy that some participants (N = 87, 9.33%) identify as ethnically Asian, which may or may not impact the observed relationship between threat of COVID-19 infection and responses to the sampled national ingroups and outgroups, given that individuals of Asian descent in the United States have received disproportionate discrimination during the pandemic^38,41^. Although the sample size is underpowered to detect effects that could be preferential to individuals who identify as ethnically Asian, we had adequate power to examine our results without them in the sample. All reported results with the full sample reported below persist if individuals identifying as ethnically Asian are not included in analyses. This is true if participants are excluded if they (1) only identify as Asian or (2) at least partially identify as Asian (i.e., indicated Mixed, with indication of Asian). We also checked whether participants’ age correlated with our predictor of interest—perceived threat of COVID-19 infection—which would indicate it should be added as a covariate in our analyses. However, age was unrelated to perceived threat of COVID-19 infection (*r* = 0.028, *p* = 0.391) indicating it does not need to be examined as a covariate. Finally, given that our data was collected on two different online participant recruitment websites (mTurk and Prolific), we checked whether participants’ threat of COVID-19 infection varied as a function of whether they were recruited from mTurk vs. Prolific. An independent samples t-test indicated that participants recruited from mTurk reported greater perceived threat of COVID-19 infection than participants recruited from Prolific (Mean_mTurk_ = 26.368, SD = 4.004; Mean_Prolific_ = 25.846, SD = 3.651; *t*(889.98) = 2.066, *p* = 0.039). Thus, our linear mixed-effects models included participant pool (mTurk vs. Prolific) as a covariate to ensure observations with threat of COVID-19 infection persisted above and beyond the participant pool predictor.

## Results

### Perceived threat of COVID-19 infection and feelings of warmth

**Figure 1:**
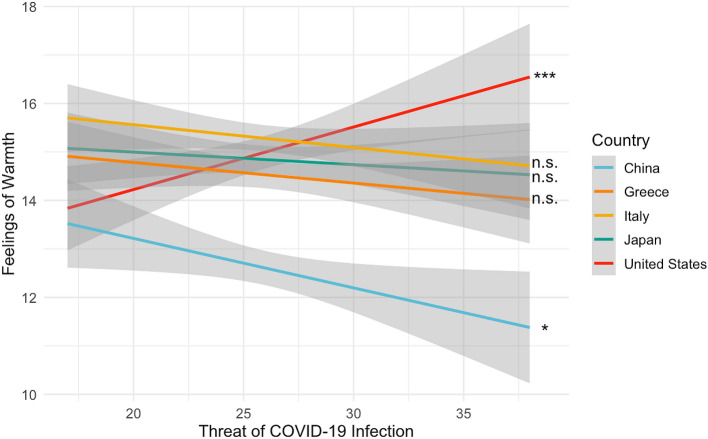
Perceived risk of COVID-19 infection is associated with greater warmth toward American nationals, less warmth toward Chinese nationals, and is unrelated to warmth felt toward all other groups considered.

Our goal was to test whether threat of COVID-19 infection relates to national ingroup preference and/or outgroup prejudice. Based on the threat-of-infection literature^12–17^, we predicted that greater perceived threat of COVID-19 infection would differentially impact participants’ warmth toward the typical American and Chinese national (given that China was communicated as the source of COVID-19), but not toward the typical individual from the other nations considered. To test this prediction, we used a linear mixed effects model to test whether participants’ risk of COVID-19 infection interacted with a “warmth contrast” of [3 -1/3 -1/3 -1/3 -2], where each regressor represents responses to the typical American, Japanese, Greek, Italian, and Chinese national, respectively. The regressor values for the countries reflect the prediction that outgroup warmth responses (relative to ingroup warmth responses) should be lower for every non-American group (hence the negative values) but more so for Chinese nationals relative to other groups (hence the greater negative value for Chinese nationals relative to the evenly distributed, less negative values for Japanese, Greek , and Italian nationals). This contrast allows us to specifically test whether the threat of COVID-19 infection modulates participants' feelings of warmth toward American and Chinese nationals in opposing directions. There was a significant interaction between the threat of COVID-19 infection and the warmth contrast (β = 0.047, *t*(3728) = 5.848, *p* < 0.001). Follow-up, correlation analyses demonstrated the direction of these effects (Fig. 1). Specifically, greater perceived threat of COVID-19 infection significantly correlated with greater warmth toward the typical American national (*r* = 0.094, *p* = 0.004) and less warmth toward the typical Chinese national (*r* = − 0.071, *p* = 0.030). Threat of COVID-19 infection was unrelated to responses to the typical Greek (*r* = − 0.038, *p* = 0.250), Japanese (*r* = − 0.022, *p* = 0.496), and Italian national (*r* = 0.043, *p* = 0.191). Follow-up paired sample t-tests showed that participants reported, on average, significantly less warmth to the typical Chinese national relative to all other countries (*t*’s > 10.99, p’s < 0.0001; See Table 1 for means and standard deviations). It is notable that the difference in warmth, on average, toward the typical Italian and American national was not significant (*t*(1776) = 1.1992 *p* = 0.2306; Table 1).

**Table 1:** Average warmth and coldness ratings for individuals from each country assessed.

Social target (perceptions of the “typical” member of each group)	Warmth felt toward target group Mean (SD)	Coldness felt toward target group Mean (SD)
American	15.011 (5.259)	2.645 (1.375)
Chinese	12.595 (5.495)	2.472 (1.477)
Japanese	14.839(4.445)	2.194 (1.332)
Italian	15.275(4.205)	2.176 (1.499)
Greek	14.524(4.311)	2.150 (1.491)

Participants reported the least warmth and greatest coldness toward Chinese nationals, of any group. Each field reports the mean and standard deviation of warmth and coldness ratings for each country.

Given that the significant correlations between threat of COVID-19 infection and warmth ratings pertained to perceptions of the typical American and Chinese national, we next sought to determine whether warmth ratings for the two countries diverged at high, medium and/or low levels of threat of COVID-19 infection. To this end, we compared the relationship between threat of infection and warmth ratings (for American vs Chinese national) among participants with threat of infection scores at least 1 standard deviation above, below, or within the mean of threat of COVID-19 infection. This analysis demonstrated that when the threat of COVID-19 infection is high, there is significantly greater warmth felt toward the typical American vs. typical Chinese national (*F*(1,930) = 8.657, p = 0.003). In contrast, when the threat of COVID-19 infection is medium or low, there is no significant difference between warmth ratings of the typical American vs. typical Chinese national (*F*_*medium*_(1,930) = 1.336 *p* = 0.248; *F*_*low*_(1,930) = 1.336 *p* = 0.248). In other words, the discrepancy in warmth felt toward American vs. Chinese nationals occurs specifically when threat of COVID-19 infection is high. Perceived vulnerability to disease (PVD) scores did not interact with the warmth contrast (β = − 0.008, *t*(3728) = − 0.049, *p* = 0.961).

Next, we assessed our prediction that trait-level prejudice moderates the relationship between threat of COVID-19 infection and ingroup preference/outgroup prejudice. An initial hint toward this possibility was the observation that trait prejudice was linked both to (1) individual threat of COVID-19 (*r* = 0.100, *p* = 0.002) and (2) interacted with the warmth contrast when included in the model (β = 0.0360, *t*(3728) = 15.917, *p* < 0.001), such that greater trait prejudice correlated with less warmth felt toward the typical Chinese national (*r* = − 0.078, *p* = 0.018) and greater warmth toward the typical American national (*r* = 0.314, *p* < 0.001).

**Figure 2:**
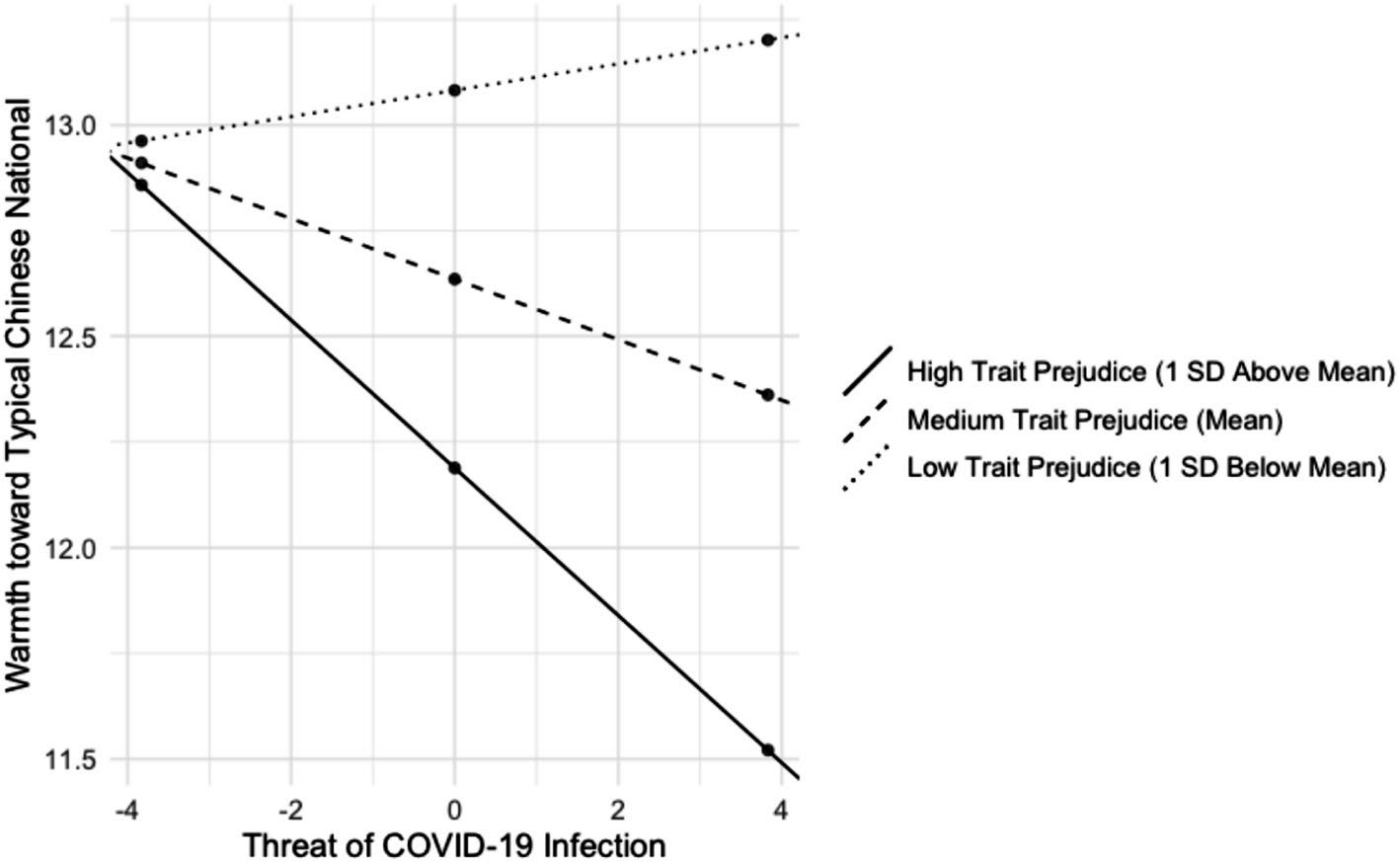
Results from the moderation analysis assessing whether trait-level prejudice impacts the relationship between threat of COVID-19 infection and warmth toward the typical Chinese national. The plotted lines reflect the simple slopes (i.e., the regression of warmth toward Chinese nationals on individual threat of COVID-19 infection) at one standard deviation above the mean of trait prejudice (solid), one standard deviation below the mean of trait prejudice (dotted), and at the mean of trait prejudice (dashed). Participants who scored low and medium on trait-level prejudice do not show a significant relationship between threat of COVID-19 infection and warmth toward the typical Chinese national. In contrast, individuals high on trait prejudice show a significant, negative relationship between threat of COVID-19 infection and warmth toward the typical Chinese national.

Indeed, moderation analyses showed that the relationship between threat of COVID-19 infection and warmth toward the typical Chinese national was moderated by trait-level prejudice (β =  − 0.014, *t* = 2.160, *p* = 0.031, [− 0.027, − 0.001]), such that participants with greater trait-level prejudice (i.e., more than 1 standard deviation above the mean) showed a significant, negative relationship between threat of COVID-19 infection and warmth toward the typical Chinese national (β = − 0.175, *t* = 2.887, *p* = 0.004, [− 0.293, − 0.056]) whereas participants medium and low on trait-prejudice (i.e., at the mean or less than 1 standard deviation above the mean) did not (medium prejudice: β = − 0.072, *t* = 1.465, *p* = 0.143, [− 0.168, 0.024]; low prejudice: β = 0.031, *t* = 0.415, *p* = 0.678, [− 0.116, 0.179]; Fig. 2). The relationship between threat of COVID-19 infection and warmth toward the typical American national was not moderated by trait-level prejudice (β = − 0.004, *t* = 0.723, *p* = 0.470 [− 0.016, 0.008]).

### Perceived threat of COVID-19 infection and feelings of coldness

**Figure 3:**
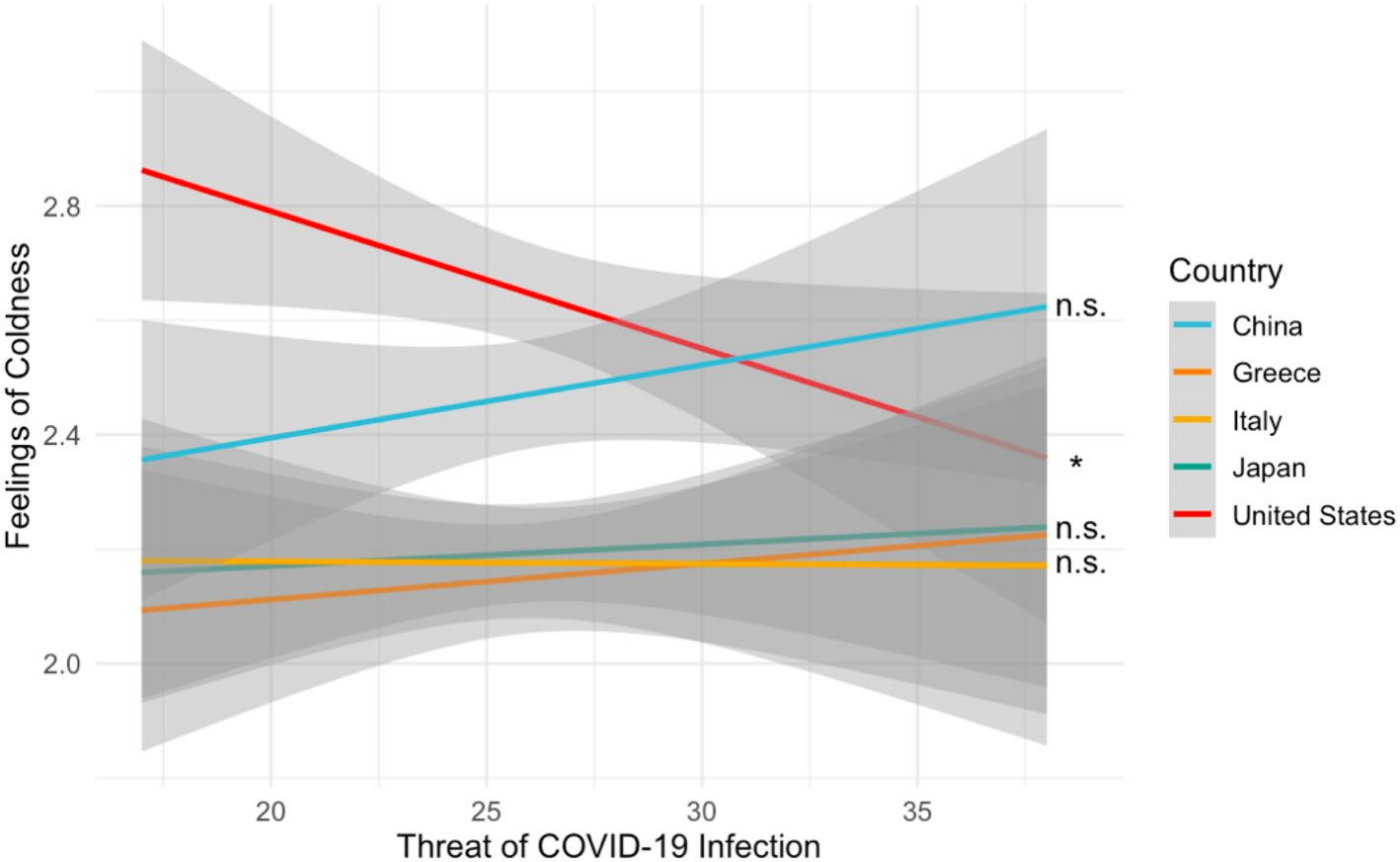
Perceived individual risk is associated with less coldness toward American nationals and is unrelated to warmth felt toward all other groups considered.

Next, we assessed the prediction that greater perceived threat of COVID-19 infection would differentially impact participants’ feelings of coldness toward the typical American and Chinese national, but not toward the typical individual from the other nations considered. Specifically, we used a linear mixed effects model to test whether participants’ risk of COVID-19 infection interacted with a “coldness contrast” of [− 3 1/3 1/3 1/3 2], where each regressor represents responses to the typical American, Japanese, Greek, Italian, and Chinese national, respectively. The regressor values for the groups reflect the prediction that outgroup coldness responses (relative to ingroup coldness responses) should be greater for every non-American group (hence the positive values) but more so for Chinese nationals relative to other outgroups (hence the more positive value for Chinese nationals relative to the evenly distributed, less positive values for Japanese, Greek, and Italian nationals). The interaction was significant (β = 0.0075, *t*(3728) = 5.758, *p* < 0.001; Fig. 3). Follow-up correlation analyses demonstrated that this interaction partially supported our prediction. That is, greater perceived individual threat of COVID-19 infection correlated with *less* coldness toward the typical American national (*r* = − 0.067, *p* = 0.042), although it was unrelated to coldness ratings of the typical Chinese national (*r* = 0.033 *p* = 0.313). Threat of COVID-19 infection was also unrelated to coldness ratings for all other countries (*r*’s < 0.016, *p*’s > 0.623).

Given that the significant correlation pertained to perceptions of the typical American national, we next sought to determine whether coldness ratings for the typical American national diverged from the other countries at high, medium and/or low levels of individual threat of COVID-19 infection. To this end, we compared the relationship between threat of infection and coldness ratings for the American nationals (vs. the average coldness rating of each other country) among participants at least 1 standard deviation above, below, or within the mean of individual threat of COVID-19 infection. This analysis demonstrated that when individual threat of COVID-19 infection is high, there is significantly less coldness felt toward the typical American national vs. nationals from the other countries (*F*(1,930) = 6.976, *p* = 0.008), and this difference is non-significant when individual threat of COVID-19 infection is medium or low (*F*_*medium*_(1,930) = 0.521, *p* = 0.471; *F*_*low*_(1,930) = 3.104, *p* = 0.078). Average trait prejudice did not moderate the relationship between individual threat of COVID-19 infection and coldness toward the typical American national (β = − 0.001, *t* = − 0.979, *p* = 0.328, [− 0.004, 0.001]). Perceived vulnerability to disease (PVD) scores also did not interact with the coldness contrast (β = -0.003, *t*(3728) = − 1.277, *p* = 0.202). It is noteworthy that as shown in Table 1, participants, on average, reported greater coldness toward the typical Chinese national relative to all other outgroups (*t*’s > 4.2752, *p*’s < 0.001), although there was the unexpected result that participants, on average, also reported greater coldness to the typical American national relative to the typical member of all other groups (*t*(185)’s > 2.6088, *p*’s < 0.009).

## Discussion

Could Americans’ fear of COVID-19 infection relate to the wave of ethnocentrism and anti-Asian prejudice observed during the pandemic? Shedding light on this question, the present results showed Americans’ perceived threat of contracting COVID-19 corresponded with less warmth toward Chinese nationals and greater warmth toward American nationals. In contrast, threat of COVID-19 infection was unrelated to feelings of warmth toward other national outgroups, including Italian (a group that also had a high COVID-19 caseload at the time of study completion) and Japanese nationals (a group that is geographically close to China, but not considered the source of COVID-19). Trait prejudice moderated these results, such that threat of COVID-19 infection related to less warmth towards Chinese nationals specifically among American participants high in prejudice. These findings suggest that threat of infection may specifically impact feelings toward the national outgroup associated with the source of the pandemic, among individuals inclined to prejudice. Participants' feelings of “coldness” (as opposed to “warmth”) showed a slightly different pattern of results, although were still consistent with prior work on threat of infection and ethnocentrism^15^. Specifically, threat of COVID-19 infection corresponded with less coldness felt toward the typical American national and was unrelated to coldness felt toward all other groups assessed. Overall, results suggest that the perceived threat of COVID-19 infection may increase ingroup preference and attenuate warmth toward national outgroups associated with the disease’s origin, particularly among individuals especially prone to prejudice.

The current findings complement and extend past research by clarifying how preexisting prejudice may shape xenophobic responses to a novel disease threat^12,14–17^. It is important to note that Sinophobia pervades American history and has long been used to justify discrimination, violence, and continued prejudice against Chinese as well as other Asian and Asian American communities^7^. In fact, the phrase “the double pandemic” refers to the observation that responses to the COVID-19 pandemic are interrelated with preexisting attitudes of racism^21^. Positioned within this context, results suggest that the threat of COVID-19 may have reified and intensified individuals’ preexisting prejudice against Chinese individuals. Consistent with this possibility, threat of infection corresponded with less warmth toward Chinese nationals only among individuals high in trait prejudice. Disease threat may therefore relate to outgroup prejudice specifically when prejudice against that outgroup is pre-existing.

Notably, our results suggest that, for Americans, feeling threatened by COVID-19 in particular, rather than disease threats in general, is likely to relate to prejudice against Chinese nationals during the COVID-19 pandemic. Only the measure of an individual’s perceived chance of contracting COVID-19, and not a trait measure of perceived vulnerability to disease more broadly, interacted with the warmth and coldness contrasts. Similarly, previous work examining a British sample found that during the COVID-19 pandemic, perceived vulnerability to disease was not uniquely related to prejudice toward ethnic minorities in Britain^42^. Taken together, our findings suggest that salient disease threats associated with a specific outgroup may be more likely to relate to prejudice toward that outgroup than individual differences in perceived disease vulnerability broadly construed.

Although threat of COVID-19 related to less warmth towards Chinese nationals specifically, it is noteworthy that another recent paper found perceived threat of COVID-19 positively correlated with White Americans’ desire to socially distance from both Asian-Americans and Black Americans^43^. This latter result suggests that fear of COVID-19 infection may increase prejudice towards outgroups not associated with the disease’s origin. However, in this paper, the correlation between COVID-19 threat and the desire to socially distance was stronger for responses to Asian-Americans than Black Americans and there was no ‘ingroup’ condition, making it challenging to determine whether COVID-19 threat corresponds with a desire to socially distance from everyone, independent of preferential prejudice towards certain groups. Future work can examine how the threat of COVID-19 relates to prejudice towards not only national ingroups and outgroups, but also outgroups within one’s own country, to further understand the relationship between disease threat and outgroup prejudice.

The observation that greater perceived threat of COVID-19 infection corresponded with less coldness toward the typical American national aligns with past work suggesting that disease threats amplify ethnocentrism^15^. However, it is noteworthy that, on average, participants reported feeling the coldest toward American nationals relative to all other countries assessed. One possible explanation of this finding is that it reflects a response bias, given the negativity of the items included in the “coldness” factor. For example, participants rated their hostility, contempt, and disgust felt for the typical person in the countries assessed. Participants may have been reluctant to endorse these statements for any outgroup member, given demand characteristics and/or the motivation to appear just. Consistent with this idea, the mean values of coldness ratings for each group are relatively low, indicating the possibility of a floor effect. Alternatively, participants’ relatively greater “coldness” and “warmth” ratings for the typical American national may reflect participants accessing different prototypical Americans when making their ratings. For example, within the category of the “typical American person,” participants may have tended to think of their political ingroup members when assessing feelings of warmth, but their political outgroup members when assessing feelings of coldness. Future research could pit these possibilities against one another, for example by (1) parameterizing the degree of negativity in the coldness ratings made and (2) manipulating whether participants think of the typical national ingroup or typical national outgroup member. Regardless, the observation that greater threat of COVID-19 infection correlated with reduced coldness toward the typical American national is, overall, consistent with the threat of infection literature.

Crucially, the results do not imply that prejudice is a universal or unavoidable response to threat of infection. Rather, findings demonstrate that responses to perceived threat are variable. Individuals may be more likely to react to health crises with violence and discrimination when diseases are politicized or framed in a biased manner^44^. Although COVID-19 infection is a concrete risk, perceptions of the virus are malleable. Better understanding the conditions under which infection risk is met with ethnocentrism and xenophobia is critical to encouraging health-promoting, rather than hateful, responses to future public health crises.

### Limitations

The present paper is not without limitations. First, we ran a PCA on a set of commonly used state prejudice scales, which allowed us to generate two dimensions of “warmth” and “coldness.” Although this approach offers greater validity than relying on a single scale, or running multiple comparisons for each composite scale, it does not allow for determining which subcomponents of state prejudice may be most impactful. Future work with targeted hypotheses can assess whether and how threat of COVID-19 infection relates to the subcomponents of prejudice.​​ Second, we do not have data from prior to the pandemic. As a result, it is unclear whether COVID-19 increased participants’ ethnocentrism and national outgroup prejudice from a pre-pandemic baseline. Nonetheless, the interaction between the perceived threat of contracting COVID-19 and ingroup and outgroup ratings aligns with the threat-of-infection literature: the more concerned participants are about contracting COVID-19, the more ingroup preference and outgroup prejudice they display. Third, ethnocentrism and outgroup prejudice may change over the time course of the COVID-19 outbreak specifically and pandemics more broadly, but data was collected during a narrow window of time (May 12–14 2020). One possibility is that the effects were short-term, specific to the initial stages of the pandemic when the threat of COVID-19 infection was particularly salient. Alternatively, given the protracted time course of the COVID-19 pandemic, the effects may have persisted over the course of the pandemic. Additionally, there may be non-linear, dynamic relationships between the threat of COVID-19 infection, geopolitical events, and prejudice. Future work is needed to assess how the threat of COVID-19 infection impacted and is impacting ethnocentrism and prejudice over time. Fourth, many of the reported effects sizes are relatively small and it is unclear if this reflects the possibility that (1) threat of COVID-19 only modestly impacts warmth and coldness toward ingroups and outgroups or (2) the possibility that the survey was not optimally designed to detect changes in prejudicial attitudes in response to infection threat. Last, all participants were residing in the U.S. at the time of reporting. It is unknown whether these results will generalize to individuals living in other countries. Additional research is needed to assess the threat of infection hypothesis across other regions of the world.

## Conclusions

The COVID-19 pandemic has seen a profound rise in expressed anti-Asian, particularly anti-Chinese, sentiment in the United States^3–6^. Here, we examined whether the fear of contracting COVID-19 tracks with this increase in prejudice. In a sample of individuals living in America, we found evidence that threat of COVID-19 infection preferentially corresponds with less warmth felt toward Chinese nationals, particularly among individuals high in trait prejudice. Additionally, and consistent with prior work finding that disease threats amplify ethnocentrism, the threat of contracting COVID-19 also corresponded with greater warmth and less coldness felt toward the typical American national. These results contribute to our understanding of how COVID-19, and pandemics more generally, may impact ethnocentrism and outgroup prejudice.

## Supplementary Information

Supplementary Information.

## Data Availability

The dataset generated and analyzed during the current study are available in the Open Science Framework, https://osf.io/sa4gq/.
