# Supplementary material for: American prejudice during the COVID-19 pandemic
Source: Sci Rep. 2022 Dec 24;12:22278. doi: 10.1038/s41598-022-26163-5 (PMC9789367; doi:10.1038/s41598-022-26163-5)
Supplement: Supplementary file 1 — Supplementary Information. [file 41598_2022_26163_MOESM1_ESM.docx]

American prejudice during the COVID-19 pandemic

Christina Huber, Sasha Brietzke, Tristen K. Inagaki, & Meghan L. Meyer

**Supplementary Materials**

**Scale Sample Items**

State Feelings Toward the Typical Individual in the Sampled Countries:

*Feeling Thermometer.* “How warm or cold do you feel toward the following groups? - Americans?” (scored on 0-100 scale, anchored at “very cold” and “very warm”)

*Cuddy Intergroup Emotion Scale* [*^1^*](https://paperpile.com/c/TWG6Sp/3AZW)*. “*To what extent do you feel each of the following emotions toward American people? - Contempt?” (scored on 1-5 scale, anchored at “not at all” and “a great deal”)

*Stephan Prejudice Scale* [*^2^*](https://paperpile.com/c/TWG6Sp/bNF8)*.* “Please honestly describe how you feel toward American people by indicating the degree of emotion you have toward that group. - Hostility” (scored on 0-7 scale, anchored at “I do not feel this emotion at all” and “I feel this emotion strongly”)

*Ten Item Personality Inventory* [*^3^*](https://paperpile.com/c/TWG6Sp/Glw7)*.* “Please write a number next to each statement to indicate the extent to which you agree or disagree with that statement. I see American people as: Extraverted, enthusiastic.” (scored on 1-7 scale, anchored at “disagree strongly” and “agree strongly”)

Trait Prejudice Scales:

*Ethnocentrism Scale*[*^4^*](https://paperpile.com/c/TWG6Sp/erRw)*. “*Most other cultures are backward compared to my culture.” (scored on 1-5 scale, anchored at “strongly disagree” and “strongly agree”)

*Intergroup Disgust Sensitivity Scale* [*^5^*](https://paperpile.com/c/TWG6Sp/Ii4y)*.* “I feel disgusted when people from other ethnic groups invade my personal space.” (scored on 1-7 scale, anchored at “strongly disagree” and “strongly agree”)

Perceived Threat of COVID-19:

*Perceived Risk of HIV Scale, adapted for COVID-19*[*^6^*](https://paperpile.com/c/TWG6Sp/usXo)*.* “What is your gut feeling about how likely you are to get infected with COVID-19?” (item scored on 1-7 scale, anchored at “extremely likely” and “extremely unlikely”; items in measure are scored on various scales out of 4, 5, 6, or 7, and scores are subsequently summed)

Trait Concern about Contracting Disease:

*Perceived Vulnerability to Disease Scale* [*^7^*](https://paperpile.com/c/TWG6Sp/AQAB) “I don’t like to write with a pencil someone else has obviously chewed on” and “I am more likely than the people around me to catch an infectious disease”; (scored on 1-7 scale, anchored at “strongly disagree” and “strongly agree”)

**Supplementary Table 1: Ethnic Breakdown of Participants.**

|  | **Count** |
| --- | --- |
| Asian | 87 (17 Multiracial) |
| Black/African | 156 (12 Multiracial) |
| Caucasian | 658 (35 Multiracial) |
| Hispanic/Latinx | 96 (54 Multiracial) |
| Native American | 32 (0 Multiracial) |
| Pacific Islander | 3 (3 Multiracial) |
| Other ethnicity | 9 |
| Prefer not to answer | 6 |

**Supplementary Table 2: Results of Principal Component Analysis.**

Rotation method: varimax with Kaiser normalization. Rotation converged in 3 iterations.

|  | Component | |
| --- | --- | --- |
|  | 1 | 2 |
| Feeling Thermometer* |  | .699 |
| **Human vs. Nonhuman Traits** |  |  |
| Cuddy (2007) Disgust* | .928 |  |
| Cuddy (2007) Contempt* | .894 |  |
| Cuddy (2007) Admiration* |  | .683 |
| Cuddy (2007) Pride | .635 |  |
| Cuddy (2007) Pity | .816 |  |
| Cuddy (2007) Sympathy* |  | .666 |
| Cuddy (2007) Envy* | .901 |  |
| Cuddy (2007) Jealousy* | .914 |  |
| Stephan (1998) Acceptance* |  | .812 |
| Stephan (1998) Dislike* | .917 |  |
| Stephan (1998) Hostility* | .930 |  |
| Stephan (1998) Sympathy* |  | .768 |
| Stephan (1998) Fear* | .924 |  |
| Stephan (1998) Warmth* |  | .890 |

Items with an asterisks (*) are included in the generation of the “coldness” (Component 1) and “warmth” (Component 2) scores.

1. [Cuddy, A. J. C., Fiske, S. T. & Glick, P. The BIAS map: behaviors from intergroup affect and stereotypes. *J. Pers. Soc. Psychol.* **92**, 631–648 (2007).](http://paperpile.com/b/TWG6Sp/3AZW)

2. [Stephan, W. G., Ybarra, O., Martnez, C. M., Schwarzwald, J. & Tur-Kaspa, M. Prejudice toward Immigrants to Spain and Israel: An Integrated Threat Theory Analysis. *J. Cross. Cult. Psychol.* **29**, 559–576 (1998).](http://paperpile.com/b/TWG6Sp/bNF8)

3. [Gosling, S. D., Rentfrow, P. J. & Swann, W. B. A very brief measure of the Big-Five personality domains. *J. Res. Pers.* **37**, 504–528 (2003).](http://paperpile.com/b/TWG6Sp/Glw7)

4. [Neuliep, J. W. & McCroskey, J. C. Ethnocentrism scale. *Measurement Instrument Database for the Social Science* (2013).](http://paperpile.com/b/TWG6Sp/erRw)

5. [Hodson, G. *et al.* The role of intergroup disgust in predicting negative outgroup evaluations. *J. Exp. Soc. Psychol.* **49**, 195–205 (2013).](http://paperpile.com/b/TWG6Sp/Ii4y)

6. [Napper, L. E., Fisher, D. G. & Reynolds, G. L. Development of the Perceived Risk of HIV Scale. *AIDS and Behavior* vol. 16 1075–1083 (2012).](http://paperpile.com/b/TWG6Sp/usXo)

7. [Duncan, L. A., Schaller, M. & Park, J. H. Perceived vulnerability to disease: Development and validation of a 15-item self-report instrument. *Pers. Individ. Dif.* **47**, 541–546 (2009).](http://paperpile.com/b/TWG6Sp/AQAB)
